# Structural properties of short-chain carboxylic acids and alcohols relate to the molecular and physiological response of *Salmonella enterica* in an acidic environment

**DOI:** 10.1007/s00253-025-13608-w

**Published:** 2025-10-31

**Authors:** Ker-Sin Ng, Tobias Busche, Christian Rückert-Reed, Maria Florencia Bambace, Ulrik Kræmer Sundekilde, Clarissa Schwab

**Affiliations:** 1 Department of Biological and Chemical Engineering, Aarhus University, Aarhus C, Denmark; 2 Microbial Genomics and Biotechnology, Centre for Biotechnology, Bielefeld University, Bielefeld, Germany; 3 Medical School East Westphalia-Lippe, Bielefeld University, Bielefeld, Germany; 4 Department of Food Science, Aarhus University, Aarhus C, Denmark

**Keywords:** *Salmonella*, Acid metabolism, Compound structure, Flagellar motility, Short-chain carboxylic acids, Short-chain alcohols

## Abstract

**Abstract:**

Short-chain carboxylic acids (SCCA) and short-chain alcohols (SCALC) are naturally occurring antimicrobials that contribute to the biopreservation of food fermentations. This study investigated the effect of structurally different SCCA/SCALC with two-carbon (acetic acid; phenylacetic acid; 2-phenylethanol), three-carbon (propionic acid; 3-phenylpropionic acid; 3-phenylpropanol), and three-carbon chain with an additional hydroxyl group (lactic acid; 3-phenyllactic acid; 1-phenylpropanol) on the fitness, metabolic activity and gene expression of the pathogen *Salmonella enterica* at pH 4.5. SCCA inhibited *Salmonella* at lower concentrations than SCALC with the exception of lactic acid, which was partly consumed. The presence of a phenyl group enhanced antimicrobial activity. SCCA but not SCALC increased the lag phase of *S. enterica*, and in general, acetate was formed when cell growth was reduced by 20% suggesting a negative impact on bacteria fitness. Principal component analysis and hierarchical clustering indicated distinct gene expression profiles of *S. enterica* in response to SCCA or SCALC. In the presence of certain SCCA/SCALC, *Salmonella* activated pathways related to cellular pH control, and 1,2-propanediol, propionic acid and ethanolamine metabolism that involved the formation of metabolosomes. Genes related to flagellar assembly were less expressed and mobility was lower in the presence of lactic and 3-phenyllactic acid compared to controls suggesting a compound-specific response.

**Key points:**

• *Differences in response among structurally different SCCA/SCALC at acidic condition.*

• *SCCA/SCALC stress interfered with cell growth and metabolism of acetic and propionic acid.*

• *Lactic acid prolonged the lag phase and reduced motility of Salmonella.*

**Supplementary Information:**

The online version contains supplementary material available at 10.1007/s00253-025-13608-w.

## Introduction

Short-chain carboxylic acids (SCCA) and short-chain alcohols (SCALC) are naturally occurring compounds that are characterized by carbon chain length of 1 to 6, and carboxyl (―COOH) and hydroxyl (―OH) as functional groups, respectively. SCCA are components of the plant biomass that are formed especially during phases of nutritional deficiency (Panchal et al. 2021). SCCA/SCALC are produced by microbes in fermented dairy, cereal, vegetable food and alcoholic (Park and Kim 2021). For example, food cultures of the families *Acetobacteraceae*, *Lactobacillaceae* and *Propionibacteriaceae* form acetic acid (AA), lactic acid (LA) and propionic acid (PA). 3-Phenyllactic acid (3-PL) can be derived from phenylalanine catabolism by *Lactobacillaceae* with 3-phenylpropionic acid (3PP, also referred to as hydrocinnamic acid) as an intermediate (Wu et al. 2023), while phenylacetic acid (PAA) was identified as a metabolite of members of the *Bacillaceae* (Pan et al. 2022). The SCALC 2-phenylethanol (2PEol), 1-phenylpropanol (1PPol) and 3-phenylpropanol (3PPol) naturally exist in plants (Bosse et al. 2013; Roccia et al. 2019; Ngah et al. 2022). Other than their role as flavoring and aroma compounds, SCCA and SCALC are considered as natural antimicrobials that inhibit food spoilers and pathogens and prolong food shelf-life (Liu et al. 2014; Debonne et al. 2020).

Both SCCA and SCALC are weak acids that damage the cytoplasmic membrane and denature membrane proteins (Otzen et al. 2007). The weak acid theory suggests that the antimicrobial activity of SCCA/SCALC increases with higher pK_a_ and lower pH because of a higher proportion of undissociated acids penetrating through the cell phospholipid bilayer (Ng et al. 2023). For example, the minimal inhibitory concentration to reduce growth of food-related contaminants and pathogens to 50% (MIC_50_) was significantly higher for LA (pK_a_ = 3.86) than for PA (pK_a_ = 4.88) at pH 4.8 and a lower concentration of PA was required to reduce bacteria growth at acidic pH 4.8 (MIC_50_ = 1.2–20.6 mM) compared to pH 6.5 (MIC_50_ = 7.0–39.1 mM) (Liang et al. 2021). Similarly, the SCALC ethanol had a stronger bactericidal effect at pH 3.0 compared to pH 7.0 (Casadei et al. 2001). In addition, the inhibitory activity of SCCA and SCALC increased with longer carbon chain length due to higher hydrophobicity (higher log K_ow_) (Ng et al. 2024) allowing better diffusion across the membrane barrier.

**Fig. 1:**
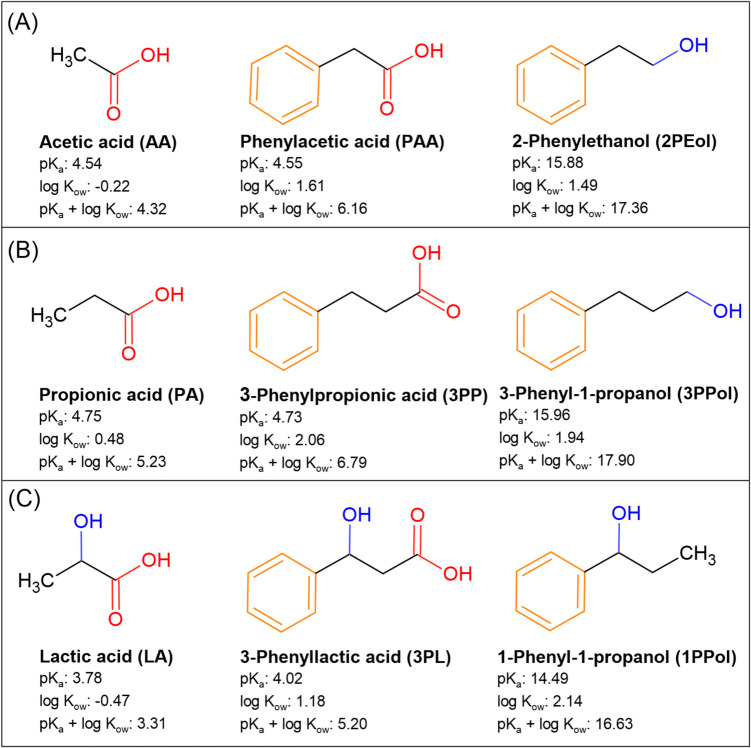
Chemical structures and physical properties of short-chain carboxylic acids (SCCA) and short-chain alcohols (SCALC) used in this study. SCCA and SCALC were classified into compounds with two-carbon chain (**A**), three-carbon chain (**B**) and three-carbon chain (**C**) with an additional hydroxyl group. The acid dissociation constant, pK_a_, and water/ethanol partition coefficient, log K_ow_, were retrieved from the Chemaxon database

When exposed to acid stress, bacteria activate a complex cellular response. Proton pumps contribute to the maintenance of pH homeostasis, membrane cyclopropane fatty acids are modified to reduce permeability towards protons, and amino acid decarboxylase activity protects against proton accumulation (Bearson et al. 2006; Lund et al. 2020). While the general acid response has been investigated for a number of food-related microbes (Álvarez-Ordóñez et al. 2012; Guan and Liu 2020; Lund et al. 2020), there have been few investigations on how structural properties of SCCA and SCALC affect antimicrobial activity and microbial response*.* To gain further mechanistic understanding on the structure–function relationship of SCCA/SCALC, the aim of this study was to investigate the impact of compositionally related SCCA and SCALC on the cellular and molecular response of *Salmonella enterica*. The genus *Salmonella* accounted for 72% of all cases of foodborne illness in Europe in 2022. *Salmonella* has been shown to survive in fermented food, e.g. in sausages and vegetable fermentations (Choi et al. 2018; Ferrer-Bustins et al. 2024), indicating the ability of *Salmonella* to encounter SCCA-related stress. We selected six SCCA (AA, PA, LA, PAA, 3PP and 3PL) and three SCALC (2PEol, 1PPol and 3PPol) with chains of two to three carbons in the backbone that contained additional phenyl and/or hydroxyl groups (Fig. 1). We determined the impact on bacterial fitness, metabolic activity and gene expression at acidic pH 4.5, which is common in fermented food.

## Materials and methods

### Strain and cultivation conditions

*Salmonella enterica* subsp. *enterica* serovar Typhimurium DSM17058 was purchased from the Deutsche Sammlung von Mikroorganismen und Zellkulturen GmbH (DSMZ, Braunschweig, Germany). Glycerol stocks (25%, v/v) were maintained at −80 °C. For reactivation, *S. enterica* was streaked on Luria Bertani (LB) (Merck) agar plates and incubated at 37 °C for 24 h at aerobic conditions. Single colonies were picked, inoculated in fresh LB broth and grown twice aerobically with 1% inoculum (v/v) at 37 °C for 24 h to obtain working cultures.

### Minimum inhibitory concentration (MIC) determination with broth dilution method

Sensitivity of *S. enterica* towards SCCA and SCALC was tested in 96-well plates using broth dilution assays. Stock solutions (100 mM) of AA, PA, LA, PAA, 3PL, 2PEol, 1PPol and 3PPol were prepared in LB broth with final pH adjustment to 4.5 with 5 N NaOH solution. The stock concentration was lower for 3PP (25 mM) due to a lower solubility. The stock solutions were filtered (0.2 μm) and stored at −20 °C until use. Acid or alcohol stock solution (100 μL) was added to 100 μL LB broth (adjusted to pH 4.5), and the two-fold dilution was performed using a PIPETMAX® automated pipetting robot (Gilson Inc.).

The final volume in each well was 100 μL, and the concentrations of SCCA or SCALC ranged from 50 to 0.1 mM, and from 12 to 0.02 mM for 3PP. Working cultures were centrifuged (4,000 rpm, 10 min), resuspended in sterile LB broth, diluted at a ratio of 1:100, and inoculated at 10% (v/v) to the wells to obtain a final cell concentration ~ 5 log CFU mL^−1^, which was estimated from plate counts of working cultures. *S. enterica* was grown without acid treatment as positive control, and a non-inoculated well that contained only broth was used as a negative control. The plates were cultivated at 37 °C for 24 h, and the OD_600_ was recorded with a microplate reader (Tecan). OD_600_ values were fitted into a four-parameter logistic regression for calculation of inflection point using GraphPad Prism 8 to determine the minimum inhibitory concentration to reduce 50% of cell population (MIC_50_) (Ng et al. 2024). Each experiment was conducted in three biologically independent replicates. Acid dissociation strength (pK_a_) was retrieved from the Chemaxon database.

### Growth assessment

Based on MIC_50_ values, suitable concentrations for each compound were selected to establish growth kinetics and calculate growth parameters. Experiments were conducted in 6-well plates at 37 °C and pH 4.5 for 24 h. The tested concentrations were 6.25–0.4 mM for PA and 3PL; 40–10 mM for LA; 6.25–0.4 mM for 3PP; and 25–3.13 mM for 1PPol and 3PPol, with a final volume of 2 mL in each well and 10% inoculum (v/v, ~ 5 log CFU mL^−1^) of *S. enterica*. The OD_600_ was recorded every 4 h and OD_600_ values were fit into a Baranyi equation to calculate the maximum growth rate (μ_max_) and lag phase (*t*_lag_) using the biogrowth R package (Garre et al. 2023). After 24 h incubation at 37 °C, 1 mL cell suspension was centrifuged (10,000 rpm, 10 min) and the supernatant was collected to assess substrate and metabolite concentrations. To investigate the AA and PA formation and/or utilization during growth, *S. enterica* was cultivated in 6-well plates at pH 4.5 with 1.56 mM of PA and 20 mM of LA. During the 36-h cultivation at 37 °C, supernatants of *Salmonella* cultures were collected (10,000 rpm, 10 min) every 4 h. Each experiment was conducted in three biologically independent replicates.

### SCCA and SCALC quantification

AA, PA and LA concentrations were measured with ultra-high performance liquid chromatography with refractive index detector (UPLC-RI) using a Vanquish Flex UPLC (Thermo Fisher Scientific). The system was equipped with an Agilent Hi-Plex H guard and main column (7.7 × 300 mm, 8 μm). Samples (10 uL) were eluted with 5 mM H_2_SO_4_ at a flow rate of 0.6 mL min^−1^ at 40 °C. 3PL, 3PP 1PPol and 3PPol were analyzed by UPLC with diode array detector (UPLC-DAD) using a 1260 Infinity II LC (Agilent Technologies). An InfinityLab Poroshell 120 EC-C18 column (3.0 × 50 mm, 2.7 μm) was used. The mobile phases were 0.1% formic acid (A) and methanol (B) at a flow rate of 0.75 mL min^−1^ with a gradient of 5% B at 0 min, 5% to 80% B from 0 to 10.67 min, 80% B from 10.67 to 13.33 min, 80% to 5% B from 13.33 to 18.67 min, and 5% B from 18.67 to 20 min at 45 °C. The DAD was set to 258 nm.

Since a medium component had same retention time as PA, we additionally used ^1^H-nuclear magnetic resonance spectrometry (NMR) as described (Buljubašić et al. 2024). Briefly, 400 μL sample was added to 200 μL sodium phosphate buffer (150 mM, containing 5 mM trimethylsilylpropanoic acid as internal shift reference) for ^1^H-NMR analysis using a Bruker Neo-IVDR 600 NMR spectrometer, operating at ^1^H frequency of 600.03 MHz and equipped with a 5 mm ^1^H BBI probe (Bruker BioSpin). ^1^D ^1^H spectrum using Bruker pulse sequence’noesygppr1d’ was acquired with 64 scans, using 4 s delay, 20 ppm spectral width, and 64 K complex data points. Chenomx NMR Suite 10.1 was used for spectra assignment (Chenomx Inc).

### Swarming test

Swarming assays were based on established methods (Irazoki et al. 2017) with modifications. Briefly, swarming plates were prepared using LB with 0.6% agar and pH adjusted to 4.5. SCCA/SCALC stock solutions were individually added to LB agar using 6-well plates (final volume 4 mL in each well) with 0.78–20 mM with concentrations used in growth assays (PA_1.56, 3PP_0.78, LA_20, 3PL_1.56, 1PPol_3.13 or 3PPol_3.13). LB agar and SCCA/SCALC solutions were mixed by circular motion and dried in the laminar flow for 30 min. *S. enterica* culture of exponential phase (OD_600_ = 0.5) was centrifuged (4,000 rpm, 10 min) and replaced with sterile 0.85% NaCl. Two microlitre of *S. enterica* suspension was inoculated in the middle of the plates, which were cultivated at 37 °C for 8 h before recording the swarming ability through measurement of the diameter of swarming zone. Each experiment was conducted in two biologically independent replicates with two technical replicates.

### RNA extraction and library preparation for RNA-seq

To investigate response in gene expression, *S. enterica* was grown at pH 4.5 in the presence of SCCA or SCALC at levels close to 0.5 × MIC_50_. Concentrations were 0.78 mM and 1.56 mM for PA (PA_0.78 and PA_1.56) and 3PL (3PL_0.78 and 3PL_1.56), 0.78 mM for 3PP (3PP_0.78), 20 mM for lactic acid (LA_20), and 3.13 mM and 6.25 mM for 1PPol (1PPol_3.13 and 1PPol_6.25) and 3PPol (3PPol_3.13 and 3PPol_6.25). *S. enterica* was cultivated in six-well plates as described above (initial inoculum ~ 5 log CFU mL^−1^). Cells were collected at exponential phase (OD_600_ 0.2–0.3 ± 0.04) after 12–20 h incubation at 37 °C, with centrifugation at 9,000 rpm, 5 min. Cell pellets were snap-frozen with liquid nitrogen and stored at −80 °C until further processing. For RNA isolation, the cell pellet was resuspended in 100 μL TE buffer containing 0.1% lysozyme, w/v), vortexed, and incubated at room temperature for 5 min. RNA was isolated with the RNeasy Mini Kit (Qiagen). The quality of RNA samples was accessed with a Bioanalyzer (Agilent Technologies) to confirm RIN > 6.0. The RIN values were not higher due to a characteristic fragmentation of 23S rRNA of *Salmonella* (Hsu et al. 1992). rRNA was removed using a riboPOOL™ rRNA depletion kit for bacteria (siTOOLs Biotech GmbH) following instructions. RNA samples were processed with Illumina Stranded Total RNA Prep with Ribo-Zero Plus kit (Illumina) to construct cDNA libraries. cDNA libraries were sequenced with the NextSeq 2000 system (P3 reagent, 100 cycles, Illumina). Approximately five million 2 × 50 nt paired-end reads were generated from each library. The Illumina TruSeq adapter sequences (34 nucleotides) were removed using *Illumina bcl2fastq2* Conversion Software v2.19.1 and standard parameters. The FASTQ files were trimmed (minimum quality 3, minimum read length to be kept 36 bp) using the Trimmomatic tool v0.39 (http://www.usadellab.org/cms/?page=trimmomatic). The processed reads were mapped to *S. enterica* subsp. *enterica* serovar Typhimurium str. LT2 reference genome (NCBI reference sequence: NC_003197.2). Transcripts were normalized according to library size using DeSeq2 R package (Bourgeois et al. 2022). Untreated controls served as the reference for comparison to samples incubated with SCCA/SCALC. Differentially expressed genes (DEGs) were identified with an absolute value of log_2_foldchange > 1 and a FDR < 0.05. DEGs were mapped to the Gene Ontology (GO) database using rbioapi R package; and to the Kyoto Encyclopedia of Genes and Genomes (KEGG) database using ClusterProfiler R package. Gene sets with FDR < 0.05 were considered as significantly enriched GO terms or KEGG pathways.

### Statistical analysis

GraphPad Prism 8 (GraphPad Software Inc.) was used to perform One-way ANOVA and Tukey’s test for multiple group comparison of the MIC_50_, μ_max_, *t*_lag_, and the SCCA/SCALC concentrations resulted from the liquid chromatography and ^1^H-NMR.

## Results

### SCCA were more inhibitory than SCALC, particularly with the presence of a phenyl group

**Fig. 2:**
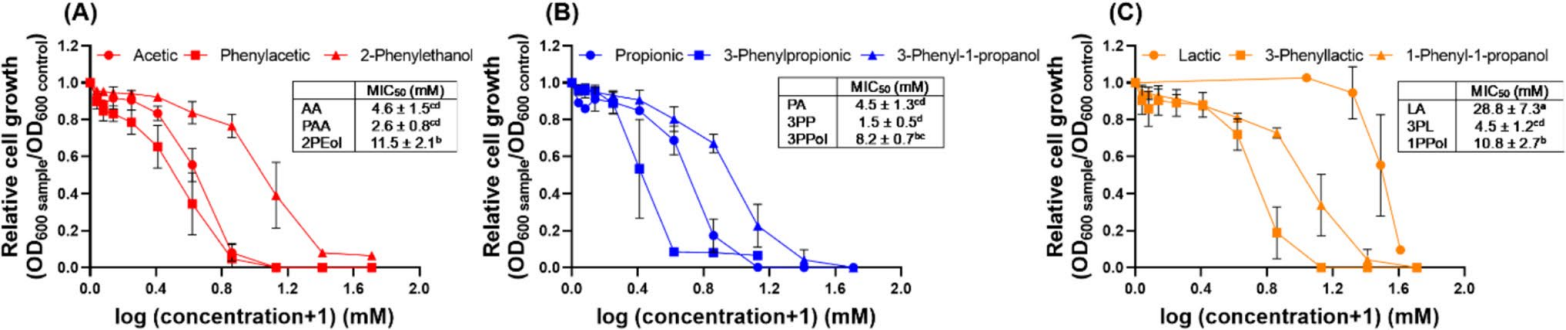
**Inhibition profiles and MIC**_**50**_** of *S. enterica* grown in the presence of SCCA and SCALC*.**** S. enterica* was grown with different concentrations of SCCA and SCALC at pH 4.5 and 37 °C for 24 h, and cell density was determined by OD_600_ measurements. The minimal inhibitory concentration to reduce density to 50% (MIC_50_) was calculated by fitting the OD_600_ values to a sigmoidal curve. (**A**-**C**) Relative cell growth of *S. enterica* in the presence of two-carbon chain (**A**), three-carbon chain (**B**) and three-carbon chain compounds with an additional hydroxyl group (**C**). Each experiment was conducted in biological triplicates

Antimicrobial activity of SCCA/SCALC against *S. enterica* DSM 17058 was tested with two-fold dilution assay in 96-well plates at pH 4.5 with concentrations ranging from 0.1–50 mM. Among the two-carbon chain compounds, PAA (MIC_50_ = 2.6 mM) and AA (MIC_50_ = 4.6 mM) were stronger antimicrobials than 2-PEol (MIC_50_ = 11.5 mM) (*P* < 0.05) (Fig. 2A). A similar pattern was observed for 3PP, PA, and 3PPol with MIC_50_ of 1.5, 4.5 and 8.2 mM respectively (Fig. 2B). 3PP conferred the strongest antimicrobial activity among the tested compounds (*P* < 0.05). LA was the least effective antimicrobial (MIC_50_ = 28.8 mM), and higher activity was observed for 3PL (MIC_50_ = 4.5 mM) and 1PPol (MIC_50_ = 10.8 mM) (Fig. 2C).

### SCCA treatments prolonged the lag phase of *S. enterica*

The capacity of cells to adapt to changes in environmental conditions is often referred to as ‘bacterial fitness’. Since two- and three-carbon chain compounds conferred similar antimicrobial activity (Fig. 2), we compared the fitness when grown in the presence of three-carbon chain compounds with and without hydroxyl group at concentrations that ranged from 0.17–0.76 fold of the MIC_50_ (Table 1).

**Table 1:** **Effect of concentrations of SCCA and SCALC on the fitness of *S. enterica*.** *S. enterica* was grown in six-well plates at 37 °C and OD_600_ was recorded in 4 h intervals for 24 h. Maximum growth rate (μ_max_) and lag phase (*t*_lag_) were calculated with Baranyi equation

	Addition of SCCA/SCALC		Growth parameters	
Compound	Concentration (mM)	Relationship of tested concentration and MIC_50_ (concentration/MIC_50_)	Lag phase *t*_lag_ (h)	Maximum growth rate μ_max_ (OD_600_*h^−1^)
Control	0	0	8.61 ± 2.28^ef^	0.17 ± 0.02^defg^
PA	0.39	0.09	**11.69 ± 0.25**^**bc**^	0.19 ± 0.01^bde^
	0.78	0.17	**12.38 ± 0.31**^**abc**^	0.20 ± 0.00^bd^
	1.56	0.35	**13.84 ± 0.43**^**ab**^	0.19 ± 0.01^bd^
3PP	0.39	0.26	**11.85 ± 0.15**^**bc**^	**0.21 ± 0.01**^**abc**^
	0.78	0.52	**13.86 ± 0.05**^**ab**^	**0.22 ± 0.01**^**ab**^
3PPol	1.56	0.19	7.34 ± 0.16^f^	0.14 ± 0.01^gh^
	3.13	0.38	8.08 ± 0.69^f^	0.15 ± 0.01^fh^
	6.25	0.76	9.13 ± 0.56^df^	0.17 ± 0.02^dh^
LA	10	0.35	**11.49 ± 0.24**^**bd**^	**0.23 ± 0.02**^**a**^
	20	0.70	**14.60 ± 0.21**^**a**^	**0.23 ± 0.00**^**ab**^
3PL	0.39	0.09	10.49 ± 0.43^cde^	0.18 ± 0.01^cdef^
	0.78	0.17	**11.22 ± 0.43**^**cd**^	0.20 ± 0.01^ad^
	1.56	0.35	**12.38 ± 0.41**^**abc**^	**0.21 ± 0.01**^**abc**^
1PPol	1.56	0.14	7.16 ± 0.05^f^	**0.14 ± 0.00**^**h**^
	3.13	0.29	7.63 ± 0.49^f^	0.14 ± 0.01^gh^
	6.25	0.58	8.75 ± 0.92^ef^	0.16 ± 0.01^eh^

^a−g^ indicate that different concentrations of a similar compound are significant differed (*P* < 0.05) within the column by Tukey’s test. The numerical value is shown in bold when there was a superscript different from the control group

Addition of SCCA prolonged the lag phase of *S. enterica* for Δ6 h (Table 1) compared to the control (*t*_lag_ = 8.6 h). The lag phase was significantly higher during growth in the presence of 0.39–1.56 mM PA (∆*t*_l*ag*_ = 3.1–5.2 h), 0.39–0.78 mM 3PP (∆*t*_l*ag*_ = 3.2–5.3 h), 0.78–1.56 mM 3PL (∆*t*_l*ag*_ = 2.6–3.8 h) and 10–20 mM LA (∆*t*_l*ag*_ = 2.9–6.0 h) (*P* < 0.05). In contrast to SCCA, SCALC had no effect on the *t*_l*ag*_ of *S. enterica* when added at < 1 × MIC_50_. For most growth conditions, the growth rate was similar to the control. The ∆μ_max_ was significantly higher when *S. enterica* was grown in the presence of 3PP (0.39–0.78 mM), 3PL (1.56 mM) and LA (10–20 mM). In contrast, growth rate was lower during growth with 1PPol (1.56 mM) (*P* < 0.05).

### *S. enterica* used certain SCCA/SCALC and produced AA and PA

**Fig. 3:**
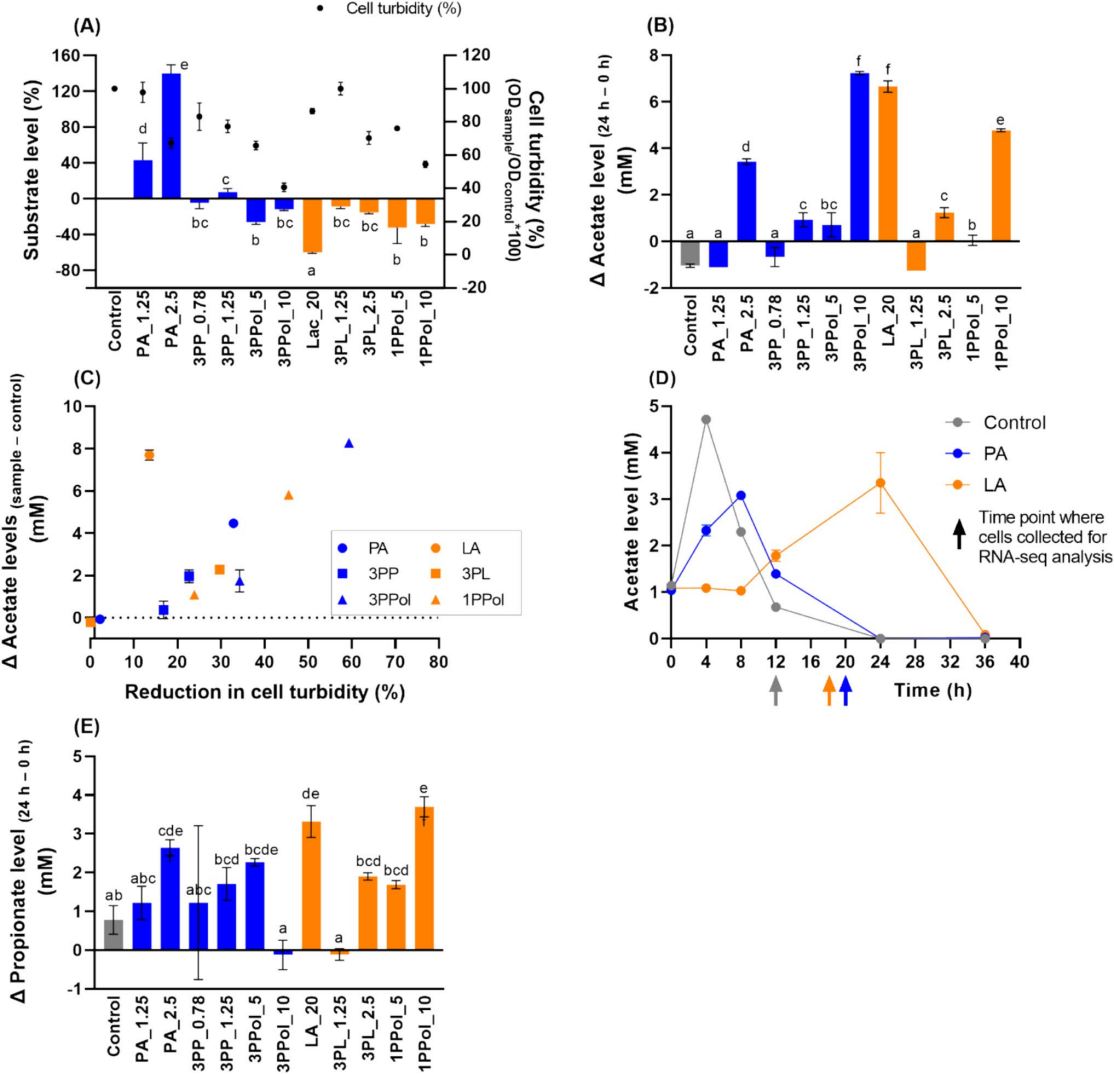
**Metabolic activity of *S. enterica* grown with SCCA/SCALC.** *S. enterica* was grown in six-well plates at concentrations less than 1 × MIC_50_ and levels of the provided SCCA/SCALC (**A**), and AA (**B**) were analyzed after incubation at 37 °C for 24 h. LA was detected with HPLC-RI, AA and PA was quantified with UPLC-RI, and 3PL, 3PP, 1PPol and 3PPol were analyzed with UPLC-DAD. Relationship of AA formation and reduction in cell turbidity (in % compared to controls) (**C**). In addition, the concentration of AA was determined every 4 h during the 36-h incubation at 37 °C (**D**). We additionally measured endpoint concentrations of PA (**E**). Multiple group comparison was conducted within different treatment groups with Tukey’s test. ^a−f^ indicate significant differences (*P* < 0.05) between SCCA/SCALC levels. Each experiment was conducted with biological triplicates

Next, we investigated the ability of *S. enterica* to metabolize the provided SCCA/SCALC and/or to produce fermentation metabolites AA and PA. *S. enterica* metabolized 60% of the provided 20 mM LA and levels of 1PPol (5–10 mM), 3PPol (5–10 mM), and 3PL (1.25–2.5 mM) were reduced by 8–32% after 24 h incubation (Fig. 3A). AA levels were significantly higher than the control when *S. enterica* was grown with 20 mM LA,10 mM 3PPol, 2.5 mM PA, and 10 mM 1PPol (3–7 mM AA) (Fig. 3B). AA was detectable (~ 1 mM) when the final turbidity was ≥ 20% lower compared to the control (Fig. 3C). There was a robust linear relationship between AA production and reduction of cell growth (R^2^ = 0.85), when the LA treatment was not included in the analysis (Fig. S1). To test for successive steps in AA metabolism, we grew *Salmonella* with PA_1.56 and LA_20 for 36 h at 37 °C. Maximal AA levels were reached later for PA_1.56 compared to controls, and AA was completely utilized at 24 h at both conditions. In the presence of LA, AA formation and utilization was delayed (Fig. 3D). PA was detected in all samples except for 1.25 mM 3PL and 10 mM 3PPol, and levels were 2–4 mM higher than in the control when *S. enterica* was grown with 2.5 mM PA, 20 mM LA and 10 mM 1PPol (Fig. 3E). Using NMR, we verified the production of PA for selected samples (Fig. S2).

### Gene expression profiles of *S. enterica* differed between SCCA and SCALC, and between SCCA with and without an additional hydroxyl side group

**Fig. 4:**
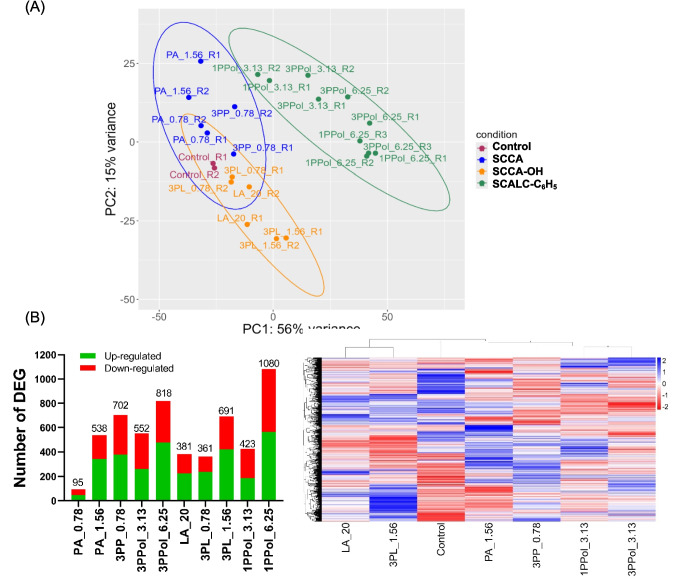
**Gene expression profiles of *S. enterica* when grown with SCCA and SCALC**. *S. enterica* was grown in six-well plates in the presence of SCCA (PA, 3PP, LA and 3PL) and SCALC (1PPol and 3PPol) with three-carbon chain at concentrations close to 0.5 × MIC_50_ at 37 °C. Cells were collected at exponential phase for RNA-seq. (**A**) Principal component analysis (PCA) of normalized gene matrix was conducted with DeSeq2 package in R, and the 95% confidence intervals for samples from similar core structures were indicated by ellipses. (**B**) Genes were characterized as differentially expressed (DEGs) if |log2foldchange|> 1 and FDR < 0.05 compared to the untreated control. (**C**) Hierarchical clustering was used to determine the dissimilarity of DEGs between treatment groups. Legend represents the row z-score

To further investigate the response of *S. enterica* against SCCA/SCALC, we determined gene expression profiles with transcriptomics when *S. enterica* was grown in the presence of SCCA/SCALC close to 0.5 × MIC_50_ at pH 4.5. In the principal component analysis (PCA), PC1 and PC2 explained 56% and 15% of the variation in gene expression profiles of *S. enterica* during SCCA/SCALC treatments (Fig. 4A). Distinct clusters illustrated that gene expression patterns differed when *Salmonella* was grown in the presence of SCALC with an additional phenyl group or at control conditions. SCCA with three-carbon chain (PA and 3PP) induced a different gene response compared to three-carbon chain compounds with an additional hydroxyl group (LA and 3PL).

When PA and 3PL were added at 1.56 mM, 538 (PA_1.56) and 691 (3PL_1.56) genes were differently expressed (DEGs) compared to controls. The number of DEGs was lower with PA_0.78 and 3PL_0.78 (95 and 361 DEGs) (Fig. 4B). Likewise, the number of DEGs was lower for 3PPol_3.13 and 1PPol_3.13 (552 and 423 DEGs) compared to 3PPol_6.25 and 1PPol_6.25 (818 and 1080 DEGs), suggesting a concentration-dependent effect on gene expression profiles.

To compare gene expression at similar stress conditions, we focussed on concentrations close to 0.3 × MIC_50_ with the exception of LA (0.7 × MIC_50_), e.g. PA_1.56, 3PP_0.78 (702 DEGs), LA_20 (381 DEGs), 3PL_1.56, 1PPol_3.13 and 3PPol_3.13. In the presence of SCCA, a bigger proportion of DEGs was upregulated (53.7–63.5%); while for SCALC, the proportion of downregulated DEG was higher (52.4–55.8%). We conducted hierarchical clustering of the 2011 DEGs from the six treatment groups (Fig. 4C) and observed that the SCALC profiles were different from SCCA; and there were differences between SCCA treatments, i.e. DEGs of LA and 3PL clustered separately from PA, 3PP and the control (Fig. 4C).

### Functions linked to DEGs differed between SCCA/SCALC

To identify the functional potential of DEGs that were affected by SCCA/SCALC-induced stressors, we mapped DEGs to the Kyoto Encyclopedia of Genes and Genomes (KEGG) and Gene Ontology (GO) databases. While the genes related to 'Metabolic Pathways’ and ‘Microbial metabolism in diverse environments’ were differential expressed in all treatments, specific pathways were differently regulated by only a few SCCA/SCALC (Table 2).

**Table 2:** **Classification of DEGs from *S. enterica* when treated with SCCA/SCALC based on KEGG.** The significantly enriched pathway (FDR < 0.05) was identified with gene set enrichment analysis. Positive and negative enrichment scores indicate that the genes were up- and down-regulated respectively in a coordinated way within a KEGG pathway

KEGG Pathway	Treatment	Enrichment Score (ES)	*P*-value	FDR
Fructose and mannose metabolism	PA_1.56	0.60	3.76E-02	9.39E-02
	3PP_0.78	0.48	4.44E-02	5.92E-02
Galactose metabolism	PA_1.56	0.61	2.79E-02	8.37E-02
Purine metabolism	1PPol_3.13	0.72	1.29E-05	7.73E-05
Lipopolysaccharide biosynthesis	3PPol_3.13	−0.59	4.96E-02	7.44E-02
Pyruvate metabolism	PA_1.56	0.62	9.48E-04	4.74E-03
	3PP_0.78	0.51	2.64E-04	4.80E-04
Propanoate metabolism	PA_1.56	0.78	2.92E-05	2.19E-04
	3PP_0.78	0.69	1.15E-06	5.73E-06
	3PL_1.56	0.56	1.98E-02	3.88E-02
Porphyrin metabolism	3PP_0.78	0.55	4.39E-04	7.32E-04
Nitrogen metabolism	3PP_0.78	0.68	1.79E-04	3.59E-04
	3PPol_3.13	0.58	5.20E-03	8.92E-03
Sulfur metabolism	LA_20	0.70	2.78E-07	2.16E-06
	3PL_1.56	0.83	1.26E-10	2.15E-09
	3PP_0.78	0.62	1.01E-06	5.73E-06
Metabolic pathways	PA_1.56	0.43	2.48E-05	2.19E-04
	3PP_0.78	0.45	9.55E-21	1.91E-19
	LA_20	0.38	2.06E-04	4.12E-04
	3PL_1.56	0.29	7.14E-04	2.43E-03
	1PPol_3.13	0.32	2.42E-04	7.26E-04
	3PPol_3.13	0.32	1.01E-07	6.05E-07
Biosynthesis of secondary metabolites	LA_20	0.40	9.03E-03	1.29E-02
	3PL_1.56	0.32	3.33E-02	4.71E-02
	3PP_0.78	0.41	1.68E-05	4.81E-05
	1PPol_3.13	0.34	7.50E-03	1.29E-02
Microbial metabolism in diverse environments	PA_1.56	0.42	2.74E-02	8.37E-02
	3PP_0.78	0.54	4.64E-14	4.64E-13
	LA_20	0.52	3.54E-05	1.18E-04
	3PL_1.56	0.45	2.45E-05	1.39E-04
	1PPol_3.13	0.60	2.03E-04	7.26E-04
	3PPol_3.13	0.57	8.75E-12	1.05E-10
Carbon metabolism	3PP_0.78	0.62	5.47E-05	1.37E-04
	3PL_1.56	0.61	2.27E-03	5.52E-03
	3PPol_3.13	0.55	1.92E-03	4.61E-03
Biosynthesis of amino acids	LA_20	0.47	2.23E-02	2.51E-02
	3PL_1.56	0.41	5.94E-02	7.77E-02
Biosynthesis of cofactors	3PP_0.78	0.31	5.88E-02	7.34E-02
Cationic antimicrobial peptide (CAMP) resistance	3PL_1.56	−0.63	9.36E-04	2.65E-03
ABC transporters	LA_20	0.59	8.65E-04	1.44E-03
	3PL_1.56	0.41	2.06E-02	3.88E-02
	3PP_0.78	0.39	2.37E-03	3.38E-03
	1PPol_3.13	0.42	1.82E-02	2.73E-02
Two-component system	LA_20	0.48	2.26E-02	2.51E-02
	3PL_1.56	0.41	2.99E-02	4.71E-02
Flagellar assembly	LA_20	−0.70	4.32E-07	2.16E-06
	3PL_1.56	−0.59	2.87E-04	1.22E-03
	3PP_0.78	0.61	1.35E-04	3.00E-04
Phosphotransferase system (PTS)	3PL_1.56	0.52	3.07E-02	4.71E-02
	3PP_0.78	0.56	7.46E-04	1.15E-03
	3PPol_3.13	0.64	1.08E-03	3.23E-03
Ribosome	LA_20	−0.61	1.03E-04	2.58E-04
	3PL_1.56	−0.66	3.28E-08	2.79E-07
	1PPol_3.13	0.58	1.97E-03	3.95E-03
Bacterial secretion system	3PP_0.78	−0.74	1.02E-05	3.41E-05
	1PPol_3.13	−0.69	7.35E-04	1.76E-03
	3PPol_3.13	−0.70	2.31E-03	4.63E-03
*Salmonella* infection	3PP_0.78	−0.75	1.83E-06	7.34E-06
	1PPol_3.13	−0.75	3.37E-06	4.04E-05
	3PPol_3.13	−0.73	8.58E-05	3.43E-04

Based on KEGG, genes related to ‘Propanoate metabolism’ were higher expressed during growth in PA_1.56, 3PP_0.78 and 3PL_1.56 (Enrichment Score (ES) = 0.56 to 0.78) (Table 2). PA_1.56 and 3PP_0.78 activated genes of ‘Fructose and mannose metabolism’ (ES = 0.48 to 0.60) and ‘Pyruvate metabolism’ (ES = 0.51 to 0.62). At the same time, genes that were associated with metabolism of 1,2-propanediol (1,2-PD) and ethanolamine, which involve in the formation of a proteinaceous microcompartment/metabolosome (Liu 2021), were upregulated when *Salmonella* was grown in the presence of PA_1.56 and 3PP_0.78.

LA_20 and 3PL_1.56 led to lower expression of genes related to ‘Flagellar assembly’ (ES = −0.59 to −0.70), and upregulated the genes associated with ‘Biosynthesis of amino acids’ (ES = 0.41 to 0.47) and ‘Sulfur metabolism’ (ES = 0.70 to 0.83); concurrently, GO assignment identified a higher expression of genes related to hydrogen sulfide biosynthesis (Fig. S3). In the presence of 3PP_0.78, 1PPol_3.13 and 3PPol_3.13, genes linked to ‘Bacterial secretion system’ and ‘*Salmonella* infection’ were downregulated (ES = −0.69 to −0.74 and −0.73 to −0.75, respectively) (Table 2), while GO identified the downregulation of type III protein secretion system with 1PPol_3.13 and 3PPol_3.13 (Fig. S3).

### *Salmonella* addressed exposure to SCCA/SCALC with upregulation of nitrate metabolism and amino acid biosynthesis

**Fig. 5:**
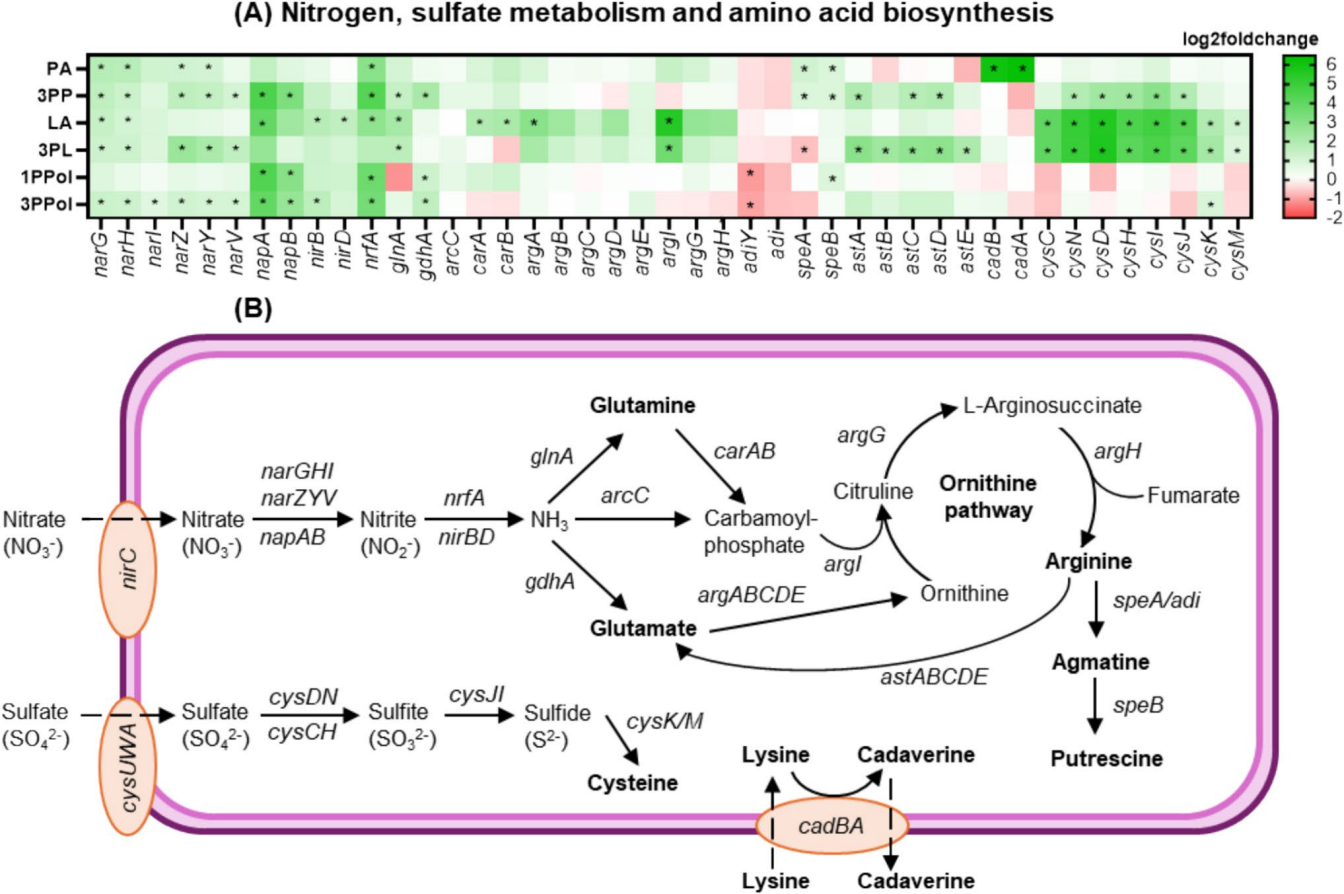
**Acid-related and general stress response of *S. enterica* when grown with SCCA and SCALC. **(**A**) Heatmap showed the log2fold change of differentially expressed genes (FDR < 0.05) relating to degradation of nitrate and sulfate, and biosynthesis of amino acids. (**B**) Schematic diagram of the associated pathways and genes for the degradation of nitrate and sulfate, and the metabolism of glutamine, glutamate, arginine, lysine and cysteine in *S. enterica*

To identify additional (acid-) related stress response, we investigated pathways encoded by differentially expressed KEGG pathways ‘Nitrogen metabolism’, ‘Sulfur metabolism’ and ‘Biosynthesis of amino acids’ in more detail (Table 2). During growth with most SCCA/SCALC, we observed upregulation of genes related to nitrogen metabolism such as *nar* and *nap* (Fig. 5A) that encode nitrate reductase, which reduces nitrate (NO_3_^−^) to nitrite (NO_2_^−^) while generating ATP (Fig. 5B). Several genes related to the formation of glutamate and arginine were higher expressed when *Salmonella* was grown with SCCA/SCALC, for example, *glnA*, *gdhA* and *arg* (Fig. 5A). *glnA* encodes glutamine synthetase that catalyzes the conversion of glutamate and NH_3_ to glutamine while *gdhA* encodes glutamate dehydrogenase for glutamate formation from α-ketoglutarate and ammonia. N-acetyl-gamma-glutamyl-phosphatereductase, acetylglutamate kinase, and argininosuccinate lyase catalyze the conversion of glutamate to arginine (Turnbull and Surette 2010); the corresponding genes *argCBH* were upregulated in the presence of LA and 3PL (Fig. 5A). Additionally, the agmatine deiminase (AgDI) pathway converts arginine into agmatine and putrescine via *speAB* and *adi* (Reitzer 2005; Papadimitriou et al. 2016); *speAB* weas upregulated during growth with PA and 3PP. When grown with PA, *cad* genes encoding lysine decarboxylase CadA, which transforms lysine to cadaverine, were upregulated. For 3PP, LA and 3PL, a significant higher expression of *cys* genes encoding sulfate transporter (CysUWA) and cysteine synthase (CysK), which reduces sulfate to cysteine, was observed.

### PA and 3PP induced genes of multiple pathways related to PA metabolism and cobalamin formation

**Fig. 6:**
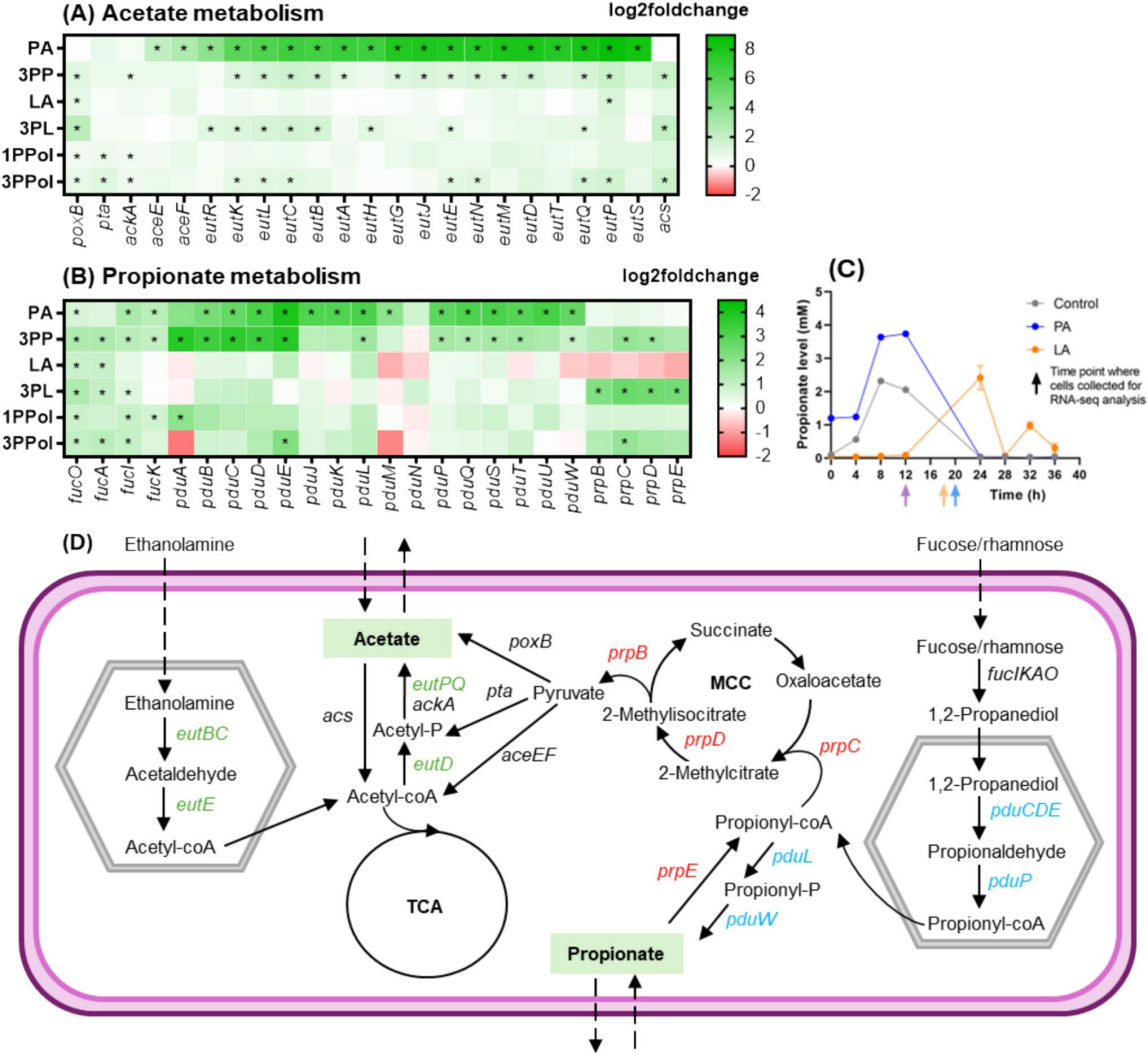
**AA and PA metabolism of *S. enterica* grown with SCCA and SCALC.** Heatmap showed the log2fold change of differentially expressed genes (FDR < 0.05) relating to AA (**A**) and PA (**B**) metabolism. Concentration of PA was determined with NMR (**C**). Schematic diagram of the associated pathways and genes for the regulation of AA and PA (**D**). The *eut* operon (green label genes) was responsible for ethanolamine degradation to produce AA, while the *pdu* (blue label genes) and *prp* (red label genes) operon were involved in 1,2-propanediol degradation and 2-methylcitrate cycle (MCC) for PA formation and consumption, respectively

We continued our investigations with a focus on the KEGG pathways ‘Pyruvate metabolism’ and ‘Propanoate metabolism’, and the GO terms related to ethanolamine and propanediol metabolism. During growth with SCCA and SCALC (except PA), *poxB* that encodes pyruvate oxidase for direct oxidation of pyruvate to AA (Starai et al. 2005; Orr et al. 2019) were upregulated (Fig. 6A). A higher expression of *pta* and *ackA* that relate to pyruvate conversion to AA via phosphotransacetylase and acetate kinase were found in 1PPol and 3PPol, while the *eutD* and *eutPQ*, which are the homologues of *pta* and *ackA*, were stimulated in the presence of PA and 3PP.

Addition of PA and 3PP activated genes related to fucose catabolism into dihydroxyacetone phosphate and L-acetaldehyde (*fucIKA*), which could be converted into 1,2-PD (*fucO*) even though we did not provide 1,2-PD, fucose or rhamnose. Concurrently, PA and 3PP activated *pdu* genes *pduCDE, pduQ, pduP PduL and pduW* (Fig. 6B), and led to upregulation of *cbi* and *cbo* genes related to cobalamin formation (Table S2). When grown with PA and 3PP, we observed the upregulation of *eutBC*, e*utE*, *eutD* and *eutPQ and genes eutSMNKL* (Fig. 6A). The regulatory protein PocR (Kim et al. 2014) regulates both *pdu* and *eut* operons but *pocR* was not differently regulated in this study (Table S2).

The *prp* genes were upregulated with 3PP, 3PL and 3PPol (Fig. 6B, 6D), and downregulated with LA. To relate gene expression to PA formation and/or utilization, we monitored PA levels during growth with PA_1.56 and LA_20 and compared to controls. With PA, higher PA levels were measured at 8 h and 12 h and PA formation and utilization was delayed with LA_20 (Fig. 6C).

### LA and 3PL reduced expression of class 2 flagellar genes and the swarming ability

**Fig. 7:**
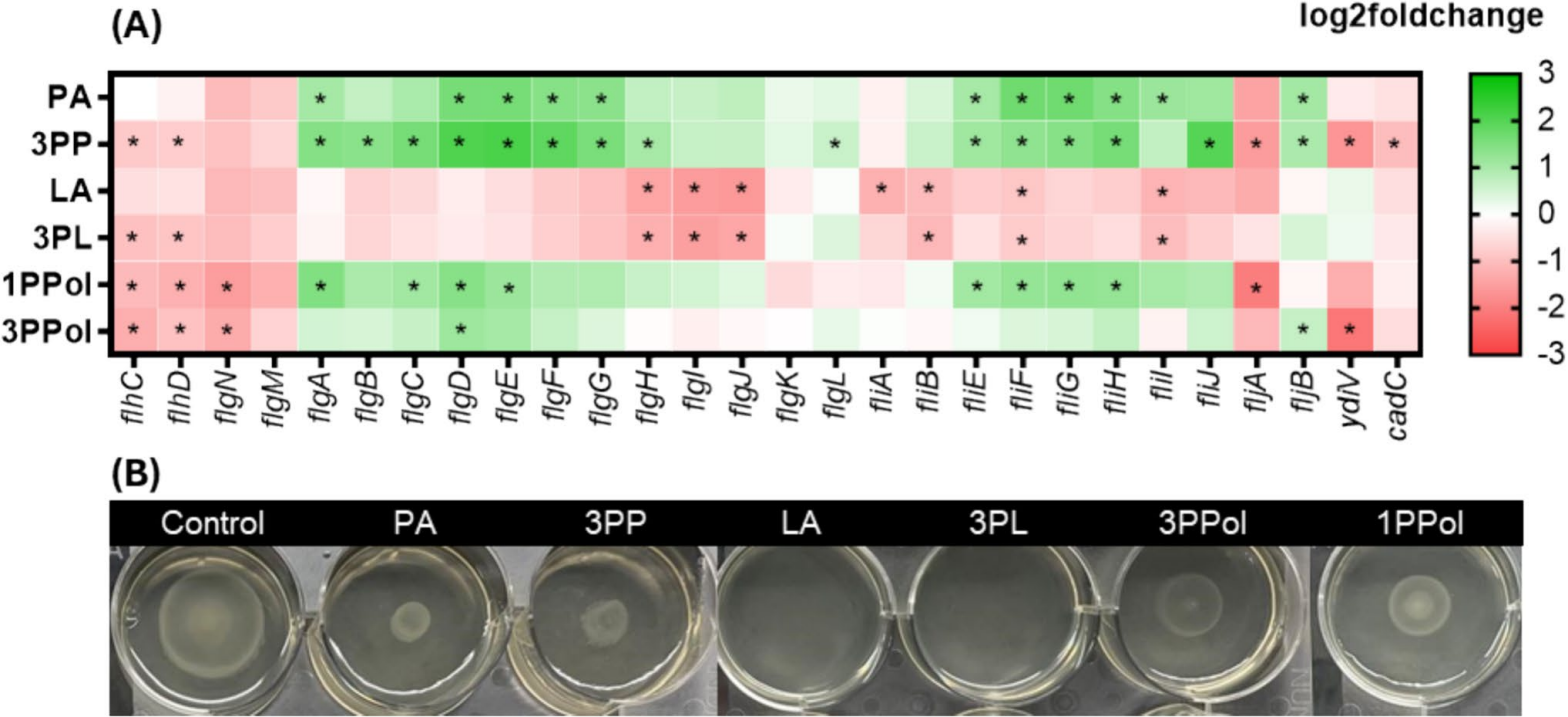
**Flagellar assembly of *S. enterica* grown with SCCA/SCALC. **(**A**) Heatmap showed the log2fold change of differentially expressed genes (FDR < 0.05) relating to flagellar activity. (**B**) Swarming motility of *S. enterica* was tested on semi-solidified LB plates (0.6% agar) that supplemented with SCCA/SCALC, all adjusted to pH 4.5. *S. enterica* (OD_600_ = 0.5) was inoculated in the middle of the plate and cultivated at 37 °C for 8 h

Flagellar formation enables *Salmonella* to relocate towards nutrient sources or escape from toxic substances and has been related to virulence (Singer et al. 2014). During cultivation of *S. enterica* with 3PP, 3PL, 1PPol and 3PPol, we observed reduced expression of *flhDC, cadC and ydiV* (Fig. 7A). LA and 3PL decreased the expression of class 2 *flg* genes, while *flgN* or *flgKL* were downregulated in the presence of 1PPol and 3PPol.

LA and 3PL prevented swarming of *S. enterica* compared to the untreated control (diameter = 2.5 cm) (Fig. 7B, Table S3), while the swarming ability was 0.3–0.5 fold lower in the presence of PA, 3PP, 1PPol and 3PPol.

## Discussion

Acid stress can be caused by low environmental pH and/or the addition of weak acids such as SCCA/SCALC. In this study, we consistently exposed *Salmonella* to acid stress as the strain was grown routinely at pH 4.5, to identify the molecular and physiological response to specific SCCA and SCALC.

Amino acid metabolism, i.e. the transformation of glutamate, lysine and arginine, has been shown to increase the acid tolerance of several taxa, including *Salmonella* (Álvarez-Ordóñez et al. 2012; Brenneman et al. 2013; Guan and Liu 2020), and related genes were also induced in this study. Cysteine formation has not been reported as a strategy of *Salmonella* related to acid resistance; however, cysteine is the precursor of glutathione that helps protecting *Salmonella* against oxidative stress (Turnbull and Surette 2010). In parallel, *nrfA* and *nirBD* that link to ammonia production (Cole and Richardson 2008) were higher expressed (Fig. 5B), which suggests that ammonium ions (NH_4_^+^) contributed to the protection of *Salmonella* during exposure to weak acids. PA and 3PP stimulated the expression of genes related to the production of biogenic amines such as agmatine, putrescine and cadaverine. These compounds can trigger allergic reaction, headache, nausea and vomiting when consumed by sensitive individuals (Li and Lu 2020). Together, these observations suggest that SCCA/SALC induced a response beyond general acid stress (Lund et al. 2020).

In this study, SCCA showed more inhibition towards *S. enterica* and had more impact on the fitness when compared to structurally similar SCALC, which was indicated by longer lag phase and delayed re-use of AA. *E. coli* and *Salmonella* are known to undergo ‘acetate switch’. During exponential growth, AA is excreted to obtain ATP, and AA can be used via acetyl-CoA in the TCA cycle in the stationary phase when the nutrients are limited (Wolfe 2005; Starai et al. 2005). In this study, we collected cells for RNA isolation at exponential phase (OD_600_ 0.2–0.3 ± 0.04), which ranged between 12 and 20 h depending on the treatment. Due to differences in sample collection points, the states of AA production/utilization were not consistent between the different treatments. LA-treated cells were collected while still producing AA, while cells grown with PA and controls had switched to utilizing AA as a carbon source. This change in timing might explain the lack of upregulation of AA metabolism genes in the LA-treated cells.

PAA, 3PL and 3PP are amphiphilic SCCA with a hydrophobic benzene ring and a hydrophilic carboxyl group. The presence of phenyl group increased the antimicrobial activity of SCCA likely due to a higher hydrophobicity, while there was little effect due to the length of carbon chain (C = 2 or 3). In a previous study, 2.5 mM 3PP destabilized the outer membrane of *S.* Typhimurium (Alakomi et al. 2007), and 3PL increased the permeability of the outer membrane of *E. coli* (Ning et al. 2017). 3PL had stronger affinity to the surface of the bacteria cell envelope compared to LA as indicated by lower zeta potential (Sorrentino et al. 2018). These observations suggest that the contribution of the phenyl group of 3PP and 3PL to antimicrobial activity was linked to interactions with the membrane. A higher antimicrobial activity was also observed on PAA, 3PL and 3PP compared to 2PEol, 1PPol and 3PPol, highlighting the contribution of the carboxyl group to inhibit *Salmonella*. Zhu et al. (2011) showed that 2PEol was less inhibitory than PAA against *E. coli*. We here report a similar mechanism for compounds 1PPol and 3PPol.

In contrast, the presence of hydroxyl group reduced antimicrobial activity, for example, LA had higher MIC_50_ values than PA, and similar observations were made for 3PL and 3PP. In a previous study, 3-hydroxypropionic acid, which is an isomer of LA, was less antimicrobial than PA against bacteria indicator strains (Liang et al. 2021). Hydroxyl-derivatives of 3PP caused lower permeability of the outer membrane of *S*. Typhimurium (Alakomi et al. 2007) possibly due to a less hydrophobic compound structure. At the same time, LA and 3PL led to the downregulation of class 2 *flg* genes, which are linked to the construction of the hook-basal body and suppressed *fliA* that facilitates the transcription of class 3 genes that are responsible for the formation of filaments and chemotaxis (Minamino et al. 2021). Initial stages of flagellum biosynthesis could be limited by lower expression of *flhDC*, *cadC* and *ydiV* (Wang et al. 2022) in 3PP, 3PL, 1PPol and 3PPol. For *Salmonella*, motility is crucial for forming and expanding bacterial colonies, particularly on surfaces of food or food processing equipment.

PA conferred antimicrobial activity, but *Salmonella* is also capable of utilizing PA with enzymes PrpBCDE to form propionyl-coA, pyruvate and succinate via the 2-methylcitrate cycle (Horswill and Escalante-Semerena 1999; Palacios et al. 2003; Yoo et al. 2017). *prp* expresion was lower when RNA was collected during growth with LA, likely because PA was still produced. *Salmonella* can also form PA from the deoxyhexoses fucose or rhamnose via the intermediate 1,2-PD by enzymes encoded by the *pdu-cbi-cob-hem* operon (Baldoma et al. 1988). *fuc* and *pdu* were co-expressed not only during anaerobic growth of *S. enterica* (Staib and Fuchs 2015), but also during aerobic growth as shown in our study. Propionyl-CoA is a shared intermediate of Pdu and PrP pathways and co-regulation of both pathways has been shown (Yoo et al. 2017).

PA also upregulated genes of the Eut operon, which catalyzes the conversion of ethanolamine to AA and is closely linked to Pdu as both pathways include the formation of metabolosomes and are dependent on cobalamin (Bobik et al. 1999), which is a co-factor for key enzymes PduCDE and EutBC (Roof and Sheppard 2014). Upregulation of *pdu* and *eut* operons by PA was observed before (Ormsby et al. 2019) and might relate to the upregulation of *cbi* and *cbo* genes. The protein PocR (Kim et al. 2014) regulates both *pdu* and *eut* operons but *pocR* was not differentially expressed in this study suggesting that other factors such as the presence of PA or pathway intermediates might be involved in the upregulation of *pdu* and *eut* operons. Furthermore, our data indicates that regulation of *pdu* and *eut* is tightly linked with *prp* and might be co-regulated by pathway intermediates and co-factors.

Food contamination and illness caused by *Salmonella* remain a major threat to the food industry. In this study, we provide critical information on the complex physiological and molecular response of *Salmonella* to natural compounds that can be used in fermented foods for biopreservation. We show that SCCA/SCALC triggered a compound-dependent response of *S. enterica* beyond the general acid stress, as all experiments were conducted at pH 4.5. We validated gene expression analysis with physiological studies to show that systems related to mobility were affected by compound of certain structures: LA and 3PL with a carboxylic and a hydroxyl group reduced flagellar motion. Our observations provide mechanistic insight into the mode of action of natural antimicrobials relevant to food biopreservation.

## Supplementary Information

Below is the link to the electronic supplementary material.

ESM1 (DOCX 642 KB)

## Data Availability

RNA-seq data was submitted to the European Nucleotide Archive (SALACID: PRJEB82633).
