# Supplementary material for: Structural properties of short-chain carboxylic acids and alcohols relate to the molecular and physiological response of Salmonella enterica in an acidic environment
Source: Appl Microbiol Biotechnol. 2025 Oct 31;109(1):240. doi: 10.1007/s00253-025-13608-w (PMC12578762; doi:10.1007/s00253-025-13608-w)
Supplement: Supplementary file 1 — (DOCX 642 KB) [file 253_2025_13608_MOESM1_ESM.docx]

**Fig. S1 Linear regression analysis of acetate production versus reduction in cell growth of *S. enterica* when grown with SCCA/SCALC.** *S. enterica* was grown in six-well plate at concentrations close to 0.5× MIC_50_, and the supernatants were collected after 24 h at 37°C. Acetate was quantified using UPLC-RI.


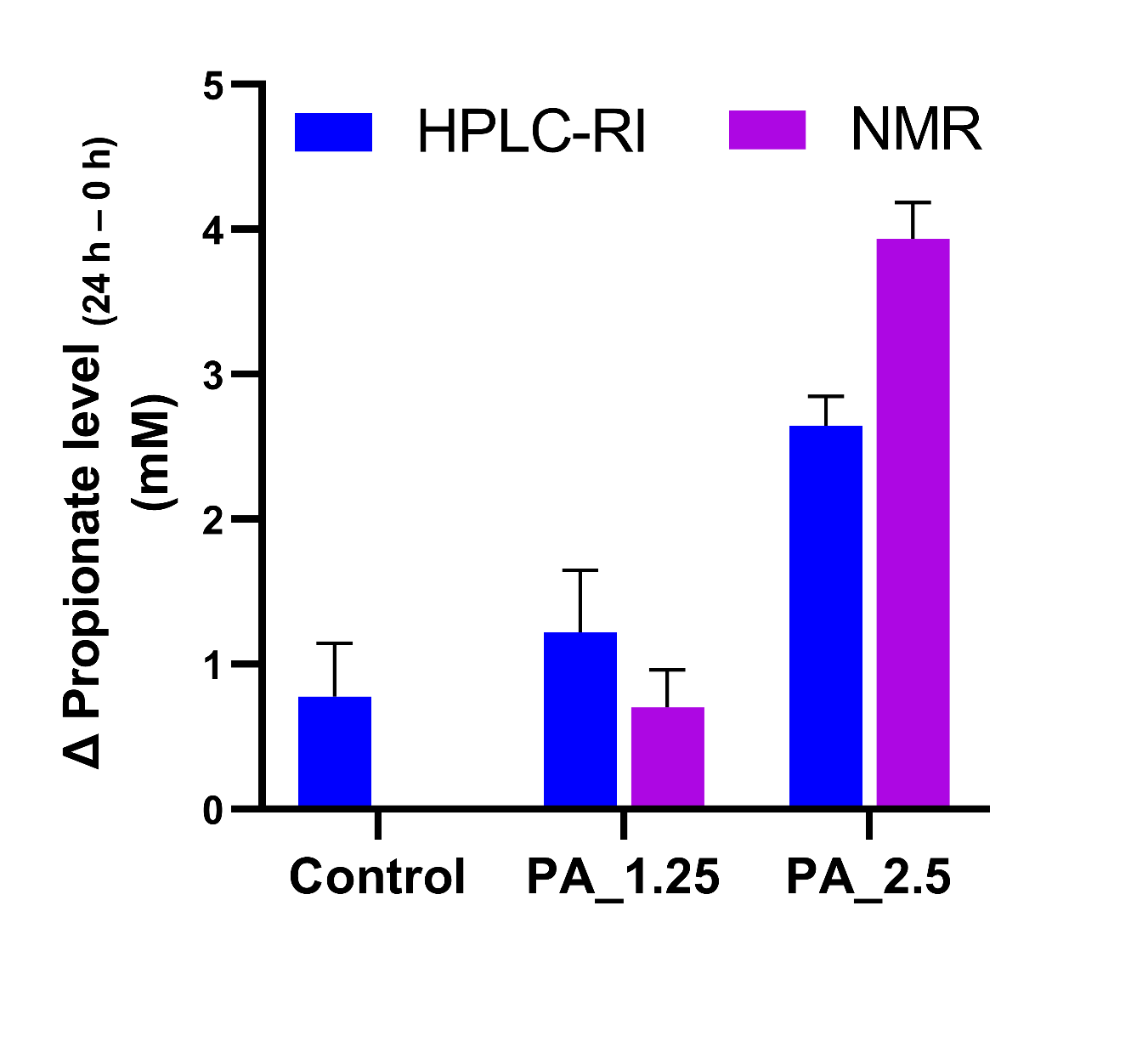


**Fig. S2 Quantification of propionate using UHPLC-RI and H^1^-NMR.** *S. enterica* was grown in six-well plates with 1.25 mM or 2.5 mM PA, and the supernatants were collected after 24 h at 37°C.


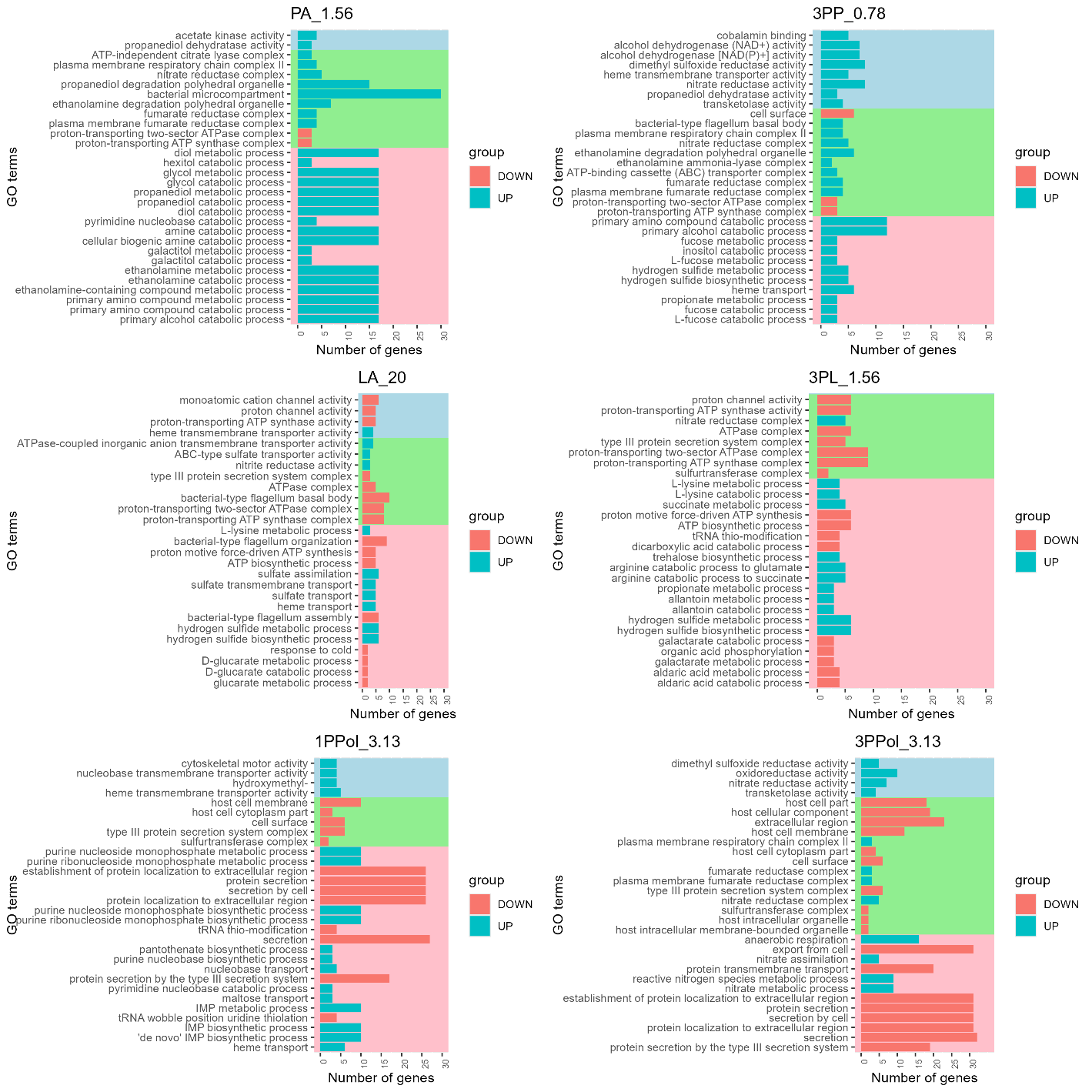


**Fig. S3 Classification of DEGs from *S. enterica* to the Gene Ontology (GO) database when grown with SCCA/SCALC.** The top 30 most enriched GO terms (FDR < 0.05) were selected with over-representation enrichment analysis. The GO terms were assigned to biological processes, cellular components and molecular function with pink, green and blue background color respectively.

| **Treated compound** | **Baranyi equation** | **R^2^** |
| --- | --- | --- |
| Acetic | Y = 0.9032/ (1 + (3.493/X)^-3.727) | 0.9941 |
| Phenylacetic | Y = 0.8558/ (1 + (2.533/X)^-2.445) | 0.9851 |
| 2-Phenylethanol | Y = 0.9397/ (1 + (10.79/X)^-2.369) | 0.9917 |
| Propionic | Y = 0.8854/ (1 + (4.311/X)^-3.820) | 0.9916 |
| 3-Phenylpropionic | Y = 0.9639/ (1 + (1.659/X)^-3.292) | 0.9926 |
| 3-Phenyl-1-propanol | Y = 0.9380/ (1 + (8.274/X)^-2.513) | 0.9925 |
| Lactic | Y = 1.0040/ (1 + (30.65/X)^-7.981) | 0.9977 |
| 3-Phenyllactic | Y = 0.8899/ (1 + (4.489/X)^-4.024) | 0.9925 |
| 1-Phenyl-1-propanol | Y = 0.9159/ (1 + (9.957/X)^-2.582) | 0.9886 |

**Table S1 Growth curve equation of *S. enterica* DSM17058 when treated with different concentrations of SCCA and SCALC compounds.** Bacterial growth was recorded with OD_600_ values (symbol Y) and fitted into the Baranyi equation using GraphPad Prism 8 software, while symbol X represents the compound concentration.

**Table S2 Differentially expressed genes (DEGs) of *S. enterica* during SCCA/SCALC treatments.** *S. enterica* was grown with SCCA/SCALC at concentrations close to 0.3× MIC_50_ (except LA at 0.7× MIC_50_) and RNA was isolated and sequenced during exponential phase. DEGs were categorized using KEGG. A log2foldchange > 1 was considered up-regulated (green background) and down-regulated (red background) when log2foldchange < -1. The numerical value is shown in bold when FDR < 0.05.

| **Locus** | **Feature** | **Gene expression (log2foldchange)** | | | | | | | **Description** |
| --- | --- | --- | --- | --- | --- | --- | --- | --- | --- |
|  |  | **PA_1.56** | **3PP_0.78** | | **LA_20** | **3PL_1.56** | **1PPol_3.13** | **3PPol_3.13** |  |
| **Nitrogen metabolism** | | | | | | | | | |
| STM1761 | narI | **1.21** | **0.82** | | **0.94** | **0.88** | 0.30 | 0.79 | nitrate reductase 1, cytochrome b(NR), gamma subunit |
| STM1762 | narJ | **1.58** | **1.14** | | 1.00 | 0.51 | 0.56 | 0.77 | nitrate reductase 1, delta subunit |
| STM1763 | narH | **1.78** | **1.32** | | **1.12** | **1.27** | 0.56 | **1.24** | nitrate reductase 1, FeS (beta) subunit |
| STM1764 | narG | **1.82** | **1.62** | | **1.44** | **1.24** | 0.83 | **1.34** | nitrate reductase 1, alpha subunit |
| STM1576 | narU | 0.38 | 0.64 | | 0.01 | **1.00** | 0.32 | 0.32 | MFS superfamily nitrate extrusion protein |
| STM1577 | narZ | **1.19** | **1.65** | | 0.70 | **2.55** | 0.66 | **1.18** | nitrate reductase 2, alpha subunit |
| STM1578 | narY | **1.12** | **1.50** | | 0.65 | **2.15** | 0.47 | **1.07** | nitrate reductase 2, beta subunit |
| STM1579 | narW | 0.65 | **1.27** | | 0.13 | **1.64** | 0.43 | **1.07** | nitrate reductase 2, delta subunit |
| STM1580 | narV | 0.49 | **1.09** | | 0.43 | **1.58** | 0.61 | **1.06** | nitrate reductase 2, gamma subunit |
| STM3474 | nirB | 0.58 | 1.38 | | **1.79** | 1.30 | 1.25 | **1.68** | nitrite reductase large subunit |
| STM3475 | nirD | 0.07 | 0.77 | | **1.55** | 1.03 | 1.20 | 1.00 | nitrite reductase small subunit |
| STM4277 | nrfA | **2.97** | **4.31** | | **2.81** | 1.72 | **3.71** | **3.51** | nitrite reductase periplasmic cytochrome c(552) |
| STM4007 | glnA | 1.08 | **1.47** | | **2.22** | **1.69** | -1.14 | 0.61 | glutamine synthetase |
| STM1299 | gdhA | 0.40 | **2.19** | | 0.45 | 0.28 | **1.27** | **1.59** | NADP-specific glutamate dehydrogenase |
| **Arginine biosynthesis and metabolism** | | | | | | | | | |
| STM4121 | argC | 0.33 | -0.03 | | 1.35 | 0.87 | -0.08 | 0.18 | N-acetyl-gamma-glutamylphosphate reductase |
| STM4122 | argB | 0.25 | 0.19 | | 2.43 | 1.30 | 0.19 | -0.02 | acetylglutamate kinase |
| STM4123 | argH | 0.44 | 0.25 | | 2.51 | 1.06 | 0.17 | -0.33 | argininosuccinate lyase |
| STM4295 | adiY | -0.33 | -0.34 | | -0.17 | -0.25 | **-1.05** | **-0.96** | transcriptional activator of adiA |
| STM4296 | adi | -0.44 | -0.47 | | 0.04 | -0.29 | -0.71 | -0.65 | arginine decarboxylase |
| STM3086 | speA | **1.15** | **0.89** | | 0.00 | **-0.65** | 0.04 | -0.61 | arginine decarboxylase |
| STM3078 | speB | **0.92** | **0.90** | | 0.42 | 0.36 | **0.88** | 0.20 | agmatinase |
| STM1303 | astC | -0.06 | **1.83** | | 0.87 | **2.78** | 0.27 | 1.23 | succinylornithine transaminase |
| STM1304 | astA | 0.14 | **2.00** | | 0.77 | **2.95** | 0.13 | 1.36 | arginine succinyltransferase |
| STM1305 | astD | 0.08 | **1.94** | | 0.68 | **2.88** | 0.21 | 1.23 | succinylglutamic semialdehyde dehydrogenase |
| STM1306 | astB | -0.43 | 1.24 | | 0.19 | **2.42** | -0.06 | 0.86 | succinylarginine dihydrolase |
| STM1307 | astE | -0.77 | 0.73 | | -0.15 | **2.08** | -0.12 | 0.40 | succinylglutamate desuccinylase |
| **Pyruvate/glycerolphospholipid metabolism** | | | | | | | | | |
| STM0935 | poxB | 0.08 | **1.04** | | **0.80** | **2.34** | 0.45 | **0.80** | pyruvate dehydrogenase/oxidase FAD and thiamine PPi cofactors, cytoplasmic in absence of cofactors |
| STM2337 | ackA | 0.36 | **0.64** | | 0.52 | 0.28 | 0.47 | **0.59** | acetate kinase A |
| STM2338 | pta | 0.72 | 0.70 | | 0.24 | 0.36 | 0.66 | **1.12** | phosphotransacetylase |
| STM2454 | eutR | **3.95** | | 0.36 | 0.17 | **1.06** | 0.27 | 0.36 | putative regulator ethanolamine operon |
| STM2455 | eutK | **5.73** | | **1.16** | 0.47 | **1.17** | 0.60 | **0.93** | putative carboxysome structural protein |
| STM2456 | eutL | **6.16** | | **1.49** | 0.35 | **1.43** | 0.53 | **0.97** | putative carboxysome structural protein |
| STM2457 | eutC | **6.72** | | **1.74** | 0.65 | **1.57** | 0.66 | **1.09** | ethanolamine ammonia-lyase, light chain |
| STM2458 | eutB | **6.58** | | **1.54** | 0.41 | **1.48** | 0.35 | 0.86 | ethanolamine ammonia-lyase, heavy chain |
| STM2459 | eutA | **6.92** | | **0.87** | 0.13 | 0.44 | 0.58 | 0.41 | CPPZ-55 prophage protein |
| STM2460 | eutH | **6.80** | | 0.60 | 0.10 | **0.97** | 0.19 | 0.15 | putative transport protein |
| STM2461 | eutG | **7.74** | | **1.12** | 0.19 | 0.56 | 0.65 | 0.15 | putative transport protein in ethanolamine utilization |
| STM2462 | eutJ | **7.90** | | **1.30** | 0.40 | 0.49 | 0.94 | 0.41 | putative heatshock protein (Hsp70) |
| STM2463 | eutE | **7.83** | | **1.14** | 0.10 | **0.76** | 0.66 | **0.95** | putative aldehyde oxidoreductase in ethanolamine utilization |
| STM2464 | eutN | **7.70** | | **1.30** | 0.48 | 0.37 | 0.66 | **1.04** | putative detox protein in ethanolamine utilization |
| STM2465 | eutM | **8.06** | | **1.21** | 0.77 | 0.64 | 1.08 | 0.62 | putative detox protein in ethanolamine utilization |
| STM2466 | eutD | **8.03** | | **1.51** | 0.57 | 0.78 | 0.90 | 1.13 | putative phosphotransacetylase in ethanolamine utilization |
| STM2467 | eutT | **7.66** | | 1.07 | 0.00 | 0.88 | 0.64 | 0.81 | putative ethanolamine utilization cobalamin adenosyltransferase |
| STM2468 | eutQ | **8.20** | | **1.41** | 0.48 | **0.91** | 0.54 | **1.40** | putative ethanolamine utilization protein |
| STM2469 | eutP | **8.72** | | **1.89** | **1.30** | 1.07 | 1.09 | **1.61** | putative ethanolamine utilization protein |
| STM2470 | eutS | **8.27** | | 1.25 | 0.38 | -0.05 | 1.09 | 0.84 | putative ethanol utilization carboxysome structural protein |
| STM4275 | acs | 0.04 | **1.85** | | 0.95 | **2.03** | 1.25 | **1.77** | acetyl-CoA synthetase |
| **Propanoate metabolism** | | | | | | | | | |
| STM0368 | prpB | 0.32 | 1.31 | | -0.64 | **2.20** | 0.69 | 1.31 | putative carboxyphosphonoenolpyruvate mutase |
| STM0369 | prpC | 0.44 | **1.84** | | -0.48 | **2.62** | 0.70 | **1.87** | putative citrate synthase |
| STM0370 | prpD | 0.32 | **1.67** | | -0.64 | **2.50** | 0.72 | 1.61 | putative protein in propionate catabolism |
| STM0371 | prpE | 0.14 | 1.43 | | -0.89 | **2.43** | 0.66 | 1.46 | putative acetyl-CoA synthetase, propionate catabolism operon |
| STM2038 | pduA | 2.07 | | **3.78** | -0.22 | -0.13 | **1.96** | -1.41 | propanediol utilization protein |
| STM2039 | pduB | **2.39** | | **3.38** | 0.48 | 0.92 | 1.54 | 0.82 | propanediol utilization protein |
| STM2040 | pduC | **2.70** | | **3.67** | 0.51 | 0.96 | 1.25 | 0.77 | propanediol utilization dehydratase, large subunit |
| STM2041 | pduD | **3.19** | | **3.45** | 0.27 | 1.07 | 1.21 | 1.34 | propanediol utilization dehydratase, medium subunit |
| STM2042 | pduE | **4.02** | | **3.79** | 1.08 | 0.10 | 1.19 | **2.21** | propanediol utilization dehydratase, small subunit |
| STM2045 | pduJ | **3.07** | | 1.19 | -0.12 | 0.42 | 0.49 | 0.57 | propanediol utilization protein |
| STM2046 | pduK | **3.05** | | 1.24 | 0.16 | -0.15 | 0.43 | 0.89 | propanediol utilization protein |
| STM2047 | pduL | **3.17** | | **1.69** | 1.12 | 0.41 | 1.10 | 1.15 | propanediol utilization protein |
| STM2048 | pduM | **2.00** | | 0.69 | -0.82 | -0.17 | 0.12 | -1.28 | propanediol utilization protein |
| STM2049 | pduN | 0.83 | | -0.15 | -0.47 | 0.77 | -0.23 | -0.12 | propanediol utilization protein |
| STM2051 | pduP | **2.83** | | **1.56** | 0.28 | 0.26 | 0.44 | 0.40 | propanediol utilization CoA-dependent propionaldehyde dehydrogenase |
| STM2052 | pduQ | **3.13** | | **1.43** | 0.33 | 0.57 | 0.69 | 0.41 | propanediol utilization propanol dehydrogenase |
| STM2053 | pduS | **3.19** | | **1.54** | 0.51 | 0.19 | 0.83 | 0.51 | propanediol utilization protein |
| STM2054 | pduT | **3.00** | | **1.58** | -0.25 | 0.61 | 0.39 | 0.86 | propanediol utilization protein |
| STM2055 | pduU | **3.27** | | 1.43 | 0.35 | 0.50 | 0.97 | 0.06 | propanediol utilization protein |
| STM2057 | pduW | **2.74** | | **0.94** | -0.56 | 0.09 | 0.33 | -0.10 | propanediol utilization propionate kinase |
| **Porphyrin metabolism** | | | | | | | | | |
| STM2016 | cobT | **1.25** | **1.14** | | 0.43 | 0.42 | 0.57 | 0.37 | nicotinate-nucleotide dimethylbenzimidazole-P phosphoribosyl transferase |
| STM2017 | cobS | **0.93** | **0.74** | | 0.11 | 0.07 | 0.06 | -0.06 | cobalamin 5'-phosphate synthase |
| STM2018 | cobU | **0.89** | **1.14** | | 0.33 | 0.05 | 0.05 | 0.44 | cobinamide kinase |
| STM2019 | cbiP | **1.19** | **1.15** | | 0.37 | 0.11 | 0.27 | 0.52 | synthesis of vitamin B12 adenosyl cobalamide precursor |
| STM2020 | cbiO | 0.61 | **0.74** | | -0.18 | -0.30 | -0.08 | 0.27 | synthesis of vitamin B12 adenosyl cobalamide precursor |
| STM2021 | cboQ | 0.86 | 0.58 | | -0.17 | -0.83 | -0.31 | -0.19 | synthesis of vitamin B12 adenosyl cobalamide precursor |
| STM2022 | cbiN | **1.11** | **1.14** | | 0.00 | 0.04 | -0.15 | 0.49 | synthesis of vitamin B12 adenosyl cobalamide precursor |
| STM2023 | cbiM | **1.56** | **1.43** | | 0.31 | 0.38 | 0.13 | 0.45 | synthesis of vitamin B12 adenosyl cobalamide precursor |
| STM2024 | cbiL | **1.98** | **1.87** | | **0.76** | **0.79** | 0.55 | 0.55 | synthesis of vitamin B12 adenosyl cobalamide precursor |
| STM2025 | cbiK | **1.61** | **1.72** | | 0.67 | 0.68 | 0.56 | 0.57 | synthesis of vitamin B12 adenosyl cobalamide precursor |
| STM2026 | cbiJ | **1.42** | **1.50** | | 0.54 | 0.62 | 0.37 | 0.46 | synthesis of vitamin B12 adenosyl cobalamide precursor |
| STM2027 | cbiH | **1.52** | **1.93** | | **0.77** | **0.75** | 0.64 | **0.86** | synthesis of vitamin B12 adenosyl cobalamide precursor |
| STM2028 | cbiG | **1.38** | **1.84** | | 0.85 | 0.53 | 0.69 | 0.51 | synthesis of vitamin B12 adenosyl cobalamide precursor |
| STM2029 | cbiF | **1.55** | **2.02** | | **0.97** | **0.81** | **0.73** | **0.87** | synthesis of vitamin B12 adenosyl cobalamide precursor |
| STM2030 | cbiT | **1.24** | **1.55** | | **0.76** | 0.08 | **0.71** | **0.83** | synthesis of vitamin B12 adenosyl cobalamide precursor |
| STM2031 | cbiE | **1.10** | **1.47** | | 0.62 | 0.26 | 0.45 | **0.68** | synthesis of vitamin B12 adenosyl cobalamide precursor |
| STM2032 | cbiD | **1.23** | **1.56** | | **0.89** | 0.57 | 0.58 | **1.01** | synthesis of vitamin B12 adenosyl cobalamide precursor |
| STM2033 | cbiC | **1.06** | **1.28** | | **0.88** | 0.06 | 0.60 | 0.68 | synthesis of vitamin B12 adenosyl cobalamide precursor |
| STM2034 | cibB | **1.37** | **1.74** | | **0.83** | 0.45 | 0.71 | 0.59 | synthesis of vitamin B12 adenosyl cobalamide precursor |
| STM2035 | cbiA | **1.68** | **1.54** | | **1.02** | **0.81** | 0.71 | **1.05** | synthesis of vitamin B12 adenosyl cobalamide precursor |
| **Flagellar assembly** | | | | | | | | | |
| STM1924 | flhC | 0.00 | **-0.85** | | -0.51 | **-0.98** | **-1.09** | **-1.28** | regulator of flagellar biosynthesis |
| STM1925 | flhD | -0.21 | **-0.80** | | -0.47 | **-0.92** | **-1.24** | **-0.96** | regulator of flagellar biosynthesis |
| STM1171 | flgN | -1.07 | -0.97 | | -1.13 | -1.06 | **-1.55** | **-1.31** | flagellar biosynthesis protein |
| STM1172 | flgM | -0.86 | -0.66 | | -1.01 | -0.80 | -1.25 | -0.70 | RflB protein |
| STM1173 | flgA | **1.05** | **1.42** | | -0.14 | -0.18 | **1.47** | 0.51 | flagellar biosynthesis protein |
| STM1174 | flgB | 0.69 | **1.37** | | -0.72 | -0.67 | 0.98 | 0.46 | flagellar biosynthesis protein |
| STM1175 | flgC | 1.00 | **1.59** | | -0.61 | -0.48 | **1.09** | 0.65 | flagellar biosynthesis protein |
| STM1176 | flgD | **1.58** | **2.05** | | -0.29 | -0.33 | **1.40** | **1.14** | flagellar biosynthesis protein |
| STM1177 | flgE | **1.60** | **2.11** | | -0.53 | -0.44 | **1.16** | 1.04 | hook protein |
| STM1178 | flgF | **1.41** | **1.88** | | -0.83 | -0.76 | 0.91 | 0.68 | flagellar biosynthesis protein |
| STM1179 | flgG | **1.35** | **1.56** | | -1.00 | -0.97 | 0.93 | 0.40 | flagellar biosynthesis protein |
| STM1180 | flgH | 0.71 | **1.03** | | **-1.40** | **-1.25** | 0.64 | -0.05 | flagellar biosynthesis protein |
| STM1181 | flgI | 0.67 | 0.68 | | **-1.57** | **-1.50** | 0.52 | -0.24 | putative flagella basal body protein |
| STM1182 | flgJ | 0.76 | 0.67 | | **-1.61** | **-1.39** | 0.36 | -0.11 | flagellar biosynthesis protein |
| STM1183 | flgK | 0.24 | 0.31 | | -0.31 | 0.07 | -0.58 | -0.02 | hook-filament junction protein 1 |
| STM1184 | flgL | 0.32 | 0.63 | | 0.05 | 0.39 | -0.31 | 0.27 | hook-filament junction protein |
| STM1954 | fliY | 0.49 | 0.56 | | **0.83** | **1.05** | 0.34 | 0.58 | putative periplasmic binding transport protein |
| STM1955 | fliZ | -0.10 | 0.06 | | **-1.11** | **-0.81** | -0.23 | 0.20 | putative regulator of FliA |
| STM1956 | fliA | -0.23 | -0.22 | | **-1.23** | -0.64 | -0.36 | 0.03 | sigma F (sigma 28) factor of RNA polymerase |
| STM1958 | fliB | 0.46 | 0.62 | | **-1.08** | **-1.13** | 0.14 | -0.11 | N-methylation of lysine residues in flagellin |
| STM1959 | fliC | 1.06 | 1.00 | | 0.11 | 0.46 | -0.16 | 0.59 | flagellar biosynthesis; flagellin |
| STM1960 | fliD | **0.75** | **1.30** | | 0.63 | **1.19** | 0.55 | **1.08** | filament capping protein |
| STM1961 | fliS | -0.05 | 0.04 | | 0.12 | **0.95** | 0.01 | 0.22 | repressor of class 3a and 3b operons (RflA activity) |
| STM1962 | fliT | -0.64 | -0.43 | | -0.71 | -0.13 | -0.61 | -0.53 | putative export chaperone for FliD |
| STM1968 | fliE | **1.03** | | **1.11** | -0.80 | -0.46 | **1.08** | 0.17 | putative flagellar hook-basal body protein |
| STM1969 | fliF | **1.68** | | **1.30** | **-0.88** | **-0.81** | **1.23** | 0.41 | basal-body MS (membrane and supramembrane)-ring and collar protein |
| STM1970 | fliG | **1.68** | | **1.41** | -0.67 | -0.68 | **1.28** | 0.47 | flagellar biosynthesis protein |
| STM1971 | fliH | **1.42** | | **1.59** | -0.77 | -0.54 | **1.24** | 0.71 | flagellar biosynthesis protein |
| STM1972 | fliI | **1.16** | | 0.71 | **-1.18** | **-1.05** | **1.03** | -0.21 | flagellum-specific ATP synthase |
| STM1973 | fliJ | 1.12 | | **1.98** | -1.13 | -0.75 | 0.92 | 0.58 | flagellar FliJ protein |
| STM1974 | fliK | **1.09** | | **1.68** | **-1.19** | **-0.97** | 0.69 | 0.28 | flagellar hook-length control protein |
| STM1975 | fliL | 0.25 | | **0.79** | **-1.35** | **-1.38** | 0.12 | -0.36 | flagellar biosynthesis |
| STM1976 | fliM | 0.19 | | 0.65 | **-1.56** | **-1.71** | 0.12 | -0.31 | flagellar biosynthesis protein |
| STM1977 | fliN | 0.39 | | 0.23 | **-1.40** | **-1.26** | 0.33 | -0.42 | flagellar biosynthesis protein |
| STM1978 | fliO | 0.21 | | 0.29 | **-1.43** | **-1.24** | 0.34 | -0.44 | flagellar biosynthesis protein |
| STM1979 | fliP | -0.19 | | -0.35 | **-1.87** | **-1.80** | -0.11 | **-1.34** | flagellar biosynthesis protein |
| STM1980 | fliQ | -0.40 | | -0.44 | **-1.55** | **-1.93** | -0.48 | **-1.47** | flagellar biosynthesis protein |
| STM1981 | fliR | -1.38 | | -1.19 | -0.65 | -1.37 | **-1.77** | **-1.69** | putative flagellar biosynthetic protein |
| STM2770 | fljA | -1.44 | **-1.57** | | -1.31 | -0.43 | **-2.04** | -1.12 | repressor of fliC |
| STM2771 | fljB | **1.07** | **0.97** | | -0.15 | 0.49 | -0.15 | **0.68** | filament structural protein |
| STM1344 | ydiV | -0.34 | **-1.68** | | 0.24 | 0.20 | -1.28 | **-2.15** | putative diguanylate cyclase/phosphodiesterase domain 1 |
| STM2557 | cadC | -0.48 | **-1.05** | | -0.53 | -0.37 | -0.27 | -0.54 | transcriptional activator of cad operon |
| **Fructose and mannose metabolism** | | | | | | | | | |
| STM2973 | fucO | **0.70** | | **1.41** | **0.94** | **1.41** | **0.90** | **1.03** | L-1,2-propanediol oxidoreductase |
| STM2974 | fucA | 0.59 | | **1.42** | **0.96** | **1.04** | 0.81 | **1.18** | L-fuculose-1-phosphate aldolase |
| STM2976 | fucI | **1.47** | | **1.21** | 0.41 | **0.55** | **0.97** | **1.02** | L-fucose isomerase |
| STM2977 | fucK | **1.23** | | **1.04** | 0.08 | 0.02 | **0.79** | 0.61 | L-fuculokinase |
| STM2978 | fucU | **0.77** | | **0.95** | -0.09 | -0.11 | 0.52 | 0.56 | conserved protein of fucose operon |
| STM2979 | fucR | 0.05 | | **1.19** | 0.15 | 0.46 | 0.57 | 0.97 | positive regulator of the fuc operon |

**Table S3 Swarming test of *S. enterica* when grown with SCCA/SCALC.** The LB with 0.6% agar was adjusted to pH 4.5. *S. enterica* (OD_600_ = 0.5) was inoculated in the middle of the plate and cultivated at 37°C for 8 h.

| **Treatment** | **Swarming diameter (cm)** | **Ratio** |
| --- | --- | --- |
| Control | 2.5 ± 0.5 | 1.0 |
| PA_1.56 | 0.9 ± 0.2 | 0.4 |
| 3PP_0.78 | 0.8 ± 0.1 | 0.3 |
| LA_20 | 0 | 0.0 |
| 3PL_1.56 | 0 | 0.0 |
| 1PPol_3.13 | 1.3 ± 0.1 | 0.5 |
| 3PPol_3.13 | 1.3 ± 0.0 | 0.5 |
